# Testing for Bacteria and Fungi in Cells and Ancillary Reagents—Probe‐Based Quantitative PCR Assays

**DOI:** 10.1111/cpr.70270

**Published:** 2026-09-02

**Authors:** Jiani Cao, Ranran Xu, Boqiang Fu, Jie Hao, Lei Wang, Aijin Ma, Qiang Wei, Hongling Zhao, Chunming Rao, Qiyuan Li, Tao Na, Shijun Hu, Lihua Zhou, Weiqi Zhang, Qubo Chen, Siming Sun, Zengshu Liu, Shuaishuai Niu, Tingting Zhang, Yuanyuan Zhang, Yidi Gan, Shuyan Wang, Glyn N. Stacey, Jun Wei, Tongbiao Zhao, Yu Alex Zhang

**Affiliations:** ^1^ Zephyrm Biotechnologies Co. Ltd. Beijing China; ^2^ Standard Committee Chinese Society for Cell Biology Shanghai China; ^3^ National Institute of Metrology Beijing China; ^4^ Beijing Institute for Stem Cell and Regenerative Medicine Beijing China; ^5^ Institute of Zoology, Institute for Stem Cell and Regeneration Chinese Academy of Sciences Beijing China; ^6^ University of Chinese Academy of Sciences Beijing China; ^7^ Beijing Technology and Business University Beijing China; ^8^ Institute of Laboratory Animal Science, Chinese Academy of Medical Sciences, Peking Union Medical College; ^9^ JOINN Pharmaceutical Quality Research and Testing (Beijing) Co. Ltd. Beijing China; ^10^ Shenzhen Medical Academy of Research and Translation Shenzhen China; ^11^ National Institutes for Food and Drug Control Beijing China; ^12^ Soochow University Suzhou China; ^13^ Biology Division National Institute of Measurement and Testing Technology Chengdu China; ^14^ China National Center for Bioinformation and Beijing Institute of Genomics Chinese Academy of Sciences Beijing China; ^15^ The Second Clinical College of Guangzhou University of Chinese Medicine Guangzhou China

## Abstract

This document specifies the technical elements for bacteria and fungi detection by PCR assay which enables rapid, broad‐spectrum detection of bacteria and fungi with high sensitivity. Qualitative judgement is based on LOD. Key detection points include efficient nucleic acid extraction, broad‐spectrum primer‐probe design, high amplification efficiency and minimised background interference.

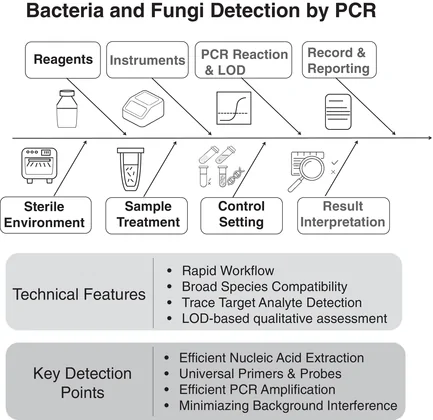

‘T/CSCB 0028‐2024 Testing for bacteria and fungi in cell preparation and ancillary materials present during the production—probe‐based quantitative PCR assay’. This standard has been jointly drafted and agreed upon by experts from the Chinese Society for Stem Cell Research. It applies to the detection of bacterial and fungal contamination in cells and ancillary reagents using probe‐based quantitative PCR assay. This document specifies the requirements for probe‐based real‐time fluorescence quantitative PCR method for detecting bacteria and fungi in cells and ancillary reagents. It includes details on the testing environment, key reagents and consumables, instrumentation and equipment, detection procedures, result interpretation, documentation and reporting, biosafety, waste disposal and contamination prevention measures. It was originally released by the *China Society for Cell Biology* on 28 October 2024. We hope that publication of the standard will facilitate the establishment of related analytical method and support therapeutic applications in stem cell and regenerative medicine.

## 1 Scope

This document specifies the requirements for using the probe‐based real‐time fluorescence quantitative PCR method to detect bacteria and fungi in cells and ancillary reagents. It includes testing environment, key reagents and consumables, instrumentation and equipment, detection procedures, result interpretation, documentation and reporting, biosafety, waste disposal and contamination prevention measures.

This standard applies to the detection of bacterial and fungal contamination in cells and ancillary reagents.

*Note:* It is important to note that there are key limitations for the effective use of 16S and 18S ribosomal DNA PCR including growth state or viability of contaminants. These are in addition to the more generic challenges for effective PCR detection; for example, PCR inhibitors in test samples, contamination with mixed species, detection of contaminants in PCR reagents and so forth. Furthermore, it is important to beae in mind that the methods reported in this standard will not detect viruses which have no ribosomes of their own.

## 2 Normative References

The following documents contain provisions that are essential to the present document. Among these, the date of the referenced document is only applicable to the version indicated; the latest version (including all amendments) of the non‐dated document is applicable to the present document.

GB/T 19489: Laboratories—General requirements for biosafety

GB/T 40365: General guide for cell sterility testing

GB/T 42077—2022/ISO 20359:2019: Biotechnology—Requirements for evaluating the performance of quantification methods for nucleic acid target sequences—qPCR and dPCR

GB/T 42753‐2023: General principles for performance evaluation of quantitative real‐time polymerase chain reaction analyser

WS/T 230: Application of polymerase chain reaction (PCR) technology in clinical diagnosis

1101 ‘Sterility Testing’ of Part IV of the *Pharmacopoeia of the People's Republic of China*

## 3 Terms and Definitions

The following terms and definitions apply to this document.

3.1 Ancillary Reagents

Reagents used in cell preparation that directly contact cells.

*Note:* For example basal media, digestive enzymes, cell cryopreservation solutions, buffers and culture supplements (e.g., serum, serum substitutes, non‐essential amino acids, growth factors, transferrin, glutamine, etc.).

## 4 Principle

Perform real‐time fluorescence PCR using universal primers and probes that target conserved sequences of bacterial 16S rRNA and fungal 18S rRNA genes. The presence of bacterial or fungal contamination in test samples can be detected based on PCR amplification curves and cycle threshold (Ct) values.

## 5 Testing Environment

Experimental procedures involving exposed samples and reagents (e.g., sample collection, nucleic acid extraction, reagent preparation and reaction setup) shall be performed in a sterile laminar flow hood, biosafety cabinet or isolator.

## 6 Key Reagents and Consumables

6.1 Experimental water shall be sterile, nuclease‐free ultrapure water complying with ISO 3696:1987 Grade 1 and ISO 22519:2023, which is sterilised (e.g., by autoclaving at 121°C for 15–30 min or 0.22 μm membrane filtration) and controlled to mitigate exogenous nucleic acid contamination.

6.2 Nucleic Acid Extraction and Purification Kit:

6.2.1 Shall be suitable for extracting bacteria and fungi from cell culture reagents, quality control samples and cell samples, and shall provide consistent pre‐treatment procedures for Gram‐positive bacteria, Gram‐negative bacteria and fungal genomic DNA extraction. Extraction reagents and consumables must be sterilised.

*Note:* The microbial lysis efficiency and nucleic acid extraction efficiency of the kit are critical factors influencing the method's limit of detection (LOD).

6.2.2 Shall be validated by a blank extraction control test using sterile, nuclease‐free ultrapure water as the sample. The Ct value of the test result shall be ≥ 30.

6.3 qPCR Master Mix shall be validated by a negative amplification test using sterile, nuclease‐free ultrapure water as the template. The Ct value of the test result should be ≥ 30.

6.4 Primers and Probes:

6.4.1 Primers and probes can be designed to target conserved regions of bacterial 16S rRNA and fungal 18S rRNA genes. Design principles, target amplicon length and specificity evaluation shall comply with Section 6.1 of *GB/T 42077‐2022/ISO 20359:2019*. Broad coverage of bacterial and fungal species shall be considered in primer/probe design. Published primer and probe sequences are provided in Appendices A and B.

6.4.2 Synthesized primers and probes shall be dissolved in sterile, nuclease‐free ultrapure water to prepare 100 μM stock solutions.

6.4.3 For multiplex PCR‐based sterility testing, use probes labelled with different fluorophores for bacterial and fungal detection.

*Note:* Fluorescence emission spectra of fluorophores typically have a normal distribution with some bandwidth overlap. When selecting multiple fluorophores, choose those with significantly different emission wavelengths to minimize cross‐channel interference.

6.5 Positive Controls

Inactivated standard strains, genomic DNA from standard strains, plasmid containing target amplification sequences or equivalent commercial products can be used as positive control.

6.6 Centrifuge tubes shall be sterile and nuclease‐free.

6.7 Pipette tips shall be sterile and nuclease‐free (sizes: 10 μL, 200 μL, 1000 μL).

## 7 Instrument and Equipment

7.1 qPCR analyser shall meet the requirements in GB/T 42753, and shall include detection channels for probe fluorescence reporter groups. qPCR analyser shall be calibrated and within the calibration validity period.

7.2 Centrifuge: Maximum relative centrifugal force (RCF) shall reach at least 20,100 × g.

7.3 All open‐sample operations shall be performed in an environment of A grade air quality (e.g., Class 2 biosafety cabinet, isolator), to assure aseptic processing and absence of lab environmental contamination.

7.4 Micropipettes (2.5, 10, 200 and 1000 μL) shall be calibrated and within the calibration validity period.

7.5 Vortex Mixer.

## 8 Detection Procedures

8.1 Precautions for Detection Operations:

The following precautions shall be observed, including but not limited to:

Personnel shall wear specific lab coats, disposable sterile latex gloves, disposable sterile non‐woven caps and medical masks.

The work area shall be sterilised and treated to remove residual nucleic acids.

Work surfaces, pipettes, centrifuges, vortex mixers and tube racks must be thoroughly disinfected using nucleic acid removal agents and 75% ethanol, followed by UV irradiation for at least 1 h.

Pipette tips, PCR tubes and centrifuge tubes must be sterilised and UV‐irradiated for at least 1 h before use.

Avoid working directly over open centrifuge tubes, tip boxes or reagent bottles.

Avoid rapid movements that may cause air turbulence.

Do not touch the inner surfaces of containers when opening centrifuge tubes, tip boxes or reagent bottles.

8.2 Sampling and Sample Pre‐Treatment

8.2.1 Sampling Volume:

The sampling volume for test samples should follow the relevant requirements of Chapter 1101 ‘Sterility Testing’ in Part IV of the Pharmacopoeia of the People's Republic of China.

8.2.2 Sampling and Sample Preparation:

Sampling and sample preparation shall meet the quality control and suitability requirements specified in the nucleic acid extraction kit instructions.

8.2.3 Sample Pre‐Treatment:

Appropriate pre‐treatment methods (e.g., propidium monoazide treatment, photochemical inactivation) can be used to reduce interference from dead bacteria and residual DNA in the samples.

8.2.4 Nucleic Acid Extraction:

Extract nucleic acids from test samples and control group samples according to the instructions provided with the nucleic acid extraction kit by the manufacturer.

8.3 Controls

8.3.1 Positive Extraction Control: Use samples containing inactivated bacterial and fungal positive controls. Process these controls in parallel with the test samples, starting from sample treatment through subsequent detection steps.

8.3.2 Blank Extraction Control: Perform extraction using sterile water or sterile buffer instead of a sample. Process this control in parallel with the test samples through subsequent detection steps.

8.3.3 Positive Amplification Control: Perform amplification using a nucleic acid solution with reliable target fragment amplification performance (e.g., bacterial and fungal genomic DNA or plasmid solutions containing the target amplification sequence).

8.3.4 Negative Amplification Control: Perform amplification using sterile water or buffer as the template.

8.4 Real‐Time Quantitative PCR Reaction

8.4.1 Prepare the reaction mixture according to the manufacturer's amplification reagent instructions. The reaction volume should be ≥ 20 μL per tube. Include ≥ 3 technical replicates per sample. Use the maximum recommended template input volume.

If 2× qPCR master mix and 50× ROX reference dye is used, example for reaction setup in Table 1 can be used as a reference.

**TABLE 1:** Reaction setup example.

Component	Volume
2× qPCR Master Mix	10 μL
Forward primer (10 μM)	0.4 μL
Reverse primer (10 μM)	0.4 μL
Probe‐based probe (10 μM)	0.2 μL
50× ROX reference dye	0.4 μL
Template	8.6 μL

*Note:* Volumes may be adjusted based on specific requirements or total reaction volume.

8.4.2 PCR Programme Parameters:

Set the real‐time fluorescence PCR reaction parameters according to the instrument specifications and amplification reagent instructions. Parameters include, but are not limited to: Reaction temperatures (denaturation, annealing, extension), ramp rates, hold times, number of amplification cycles and fluorescence acquisition settings (e.g., channel selection, read time). Number of amplification cycles shall be ≥ 40.

8.5 LOD

8.5.1 LOD Establishment:

The LOD should be determined through detection experiments using gradient spiked positive strains in test samples (for an example of an experimental protocol, refer to Appendix C).

8.5.2 Representative Strains for LOD Determination:

Select representative strains, including the positive strains specified in Chapter 1101 ‘Sterility Testing’ of Part IV of the Pharmacopoeia of the People's Republic of China and high‐risk environmental strains from the cell preparation environment.

8.5.3 Key Factors Affecting LOD include, but are not limited to:

- Type of test sample.
- Nucleic acid extraction method.
- Primer and probe sequences.
- Composition of amplification reagents.
- qPCR instrument and parameter settings.

LOD shall be re‐evaluated if any of the above factors are modified.

8.5.4 LOD shall be reported when the detection result is negative.

## 9 Result Interpretation

9.1 Quality Control

The detection experiment shall be determined to be valid only if all the following criteria are met. If any of the following criteria are not met, the detection experiment shall be determined to be invalid and shall be repeated.

- Positive Extraction Control: positive and valid amplification of the target genes and the Ct values shall be < 30.
- Positive Amplification Control: positive and valid amplification of the target genes and the Ct values shall be < 30.
- Blank Extraction Control: no detectable amplification (no typical amplification curve) or the Ct values are ≥ 30.
- Negative Amplification Control: no detectable amplification (no typical amplification curve) or the Ct values are ≥ 30.

9.2 Result Interpretation and Reporting

- Bacterial Content Below LOD: If the Ct values for the 16S target gene amplification in the test samples are all > 30, and there is no significant difference or a significant increase in Ct values compared with the blank extraction control and negative amplification control (*t*‐test, *p* > 0.05), the bacterial content in the sample can be determined to be below the LOD.
- Bacterial Content Detected: If the Ct values for the 16S target gene amplification in the test samples are all ≤ 30, or there is a significant decrease in Ct values compared with the blank extraction control and negative amplification control (*t*‐test, *p* ≤ 0.05), the sample can be determined to contain bacteria.
- Fungal Content Below LOD: If the Ct values for the 18S target gene amplification in the test samples are all > 30, and there is no significant difference or a significant increase in Ct values compared with the blank extraction control and negative amplification control (*t*‐test, *p* > 0.05), the fungal content in the sample can be determined to be below the LOD.
- Fungal Content Detected: If the Ct values for the 18S target gene amplification in the test samples are all ≤ 30, or there is a significant decrease in Ct values compared with the blank extraction control and negative amplification control (*t*‐test, *p* ≤ 0.05), the sample can be determined to contain fungi.

## 10 Documentation and Reporting

10.1 Record Requirements

Records shall include, but are not limited to, the following information:

- Sample information: Type, lot number and source descriptors.
- Detection details: Date, location and personnel performing the test.
- Instrument: Manufacturer, model and relevant settings.
- Reagents: Name, source and lot number.
- Detection procedures.
- Abnormal observations.
- Test results and the file path where raw data is stored.

10.2 Report Requirements

The report shall include, but is not limited to, the following information:

- Sample information: Type, lot number and source
- Detection details: Date, location and personnel performing the test
- Detection method and procedure.
- Reagents: Name, source and lot number.
- Instrumentation: Manufacturer, model and relevant settings.
- Test results and the LOD for the validated method.
- Abnormal observations.

## 11 Biosafety Requirements

Microbial testing shall comply with the requirements specified in GB/T 40365 and GB/T 19489.

## 12 Waste Disposal and Contamination Prevention Measures

12.1 Waste materials shall be handled in accordance with medical waste management regulations.

12.2 Contamination prevention and control measures must be implemented in compliance with WS/T 230.

## Author Contributions

Jiani Cao designed and established the method, and drafted the manuscript. Jiani Cao, Tongbiao Zhao and Yu Alex Zhang contributed to conception and design. Jiani Cao, Ranran Xu, Boqiang Fu, Jie Hao, Lei Wang, Aijin Ma, Qiang Wei and Hongling Zhao drafted and revised the manuscript. Chunming Rao, Qiyuan Li, Tao Na, Shijun Hu, Lihua Zhou, Weiqi Zhang, Qubo Chen, Siming Sun, Zengshu Liu, Shuaishuai Niu, Tingting Zhang, Yuanyuan Zhang, Yidi Gan, Shuyan Wang, Glyn N. Stacey, Jun Wei, Tongbiao Zhao and Yu Alex Zhang critically read and revised the manuscript.

## Funding

This work was supported by the National Key Research and Development Program of China (2023YFC3605100) and the Chongqing Key Technology Innovation and Application Program (CSTB2025TIAD‐STX0044).

## Data Availability

Data sharing not applicable to this article as no datasets were generated or analysed during the current study.

## Appendix A (Informative) (see Table A1)

**TABLE A1:** Representative published universal primers and probes for bacteria detection.

Primer/probe	Sequence (5′‐3′)	References
16S‐AF331^F^	TCCTACGGGAGGCAGCAGT	Determination of bacterial load by real‐time PCR using a broad‐range (universal) probe and primers set. *Microbiology*. 2002 [1]
16S‐AR797^R^	GGACTACCAGGGTATCTAATCCTGTT	
16S‐AT506^T^	CGTATTACCGCGGCTGCTGGCAC	
16S‐BF349^F^	AGGCAGCAGTDRGGAAT	Rapid detection and quantification of members of the archaeal community by quantitative PCR using fluorogenic probes. *Appl Environ Microbiol*. 2000 [2]
16S‐BR806^R^	GGACTACYVGGGTATCTAAT	
16S‐BT343^T^	TGCCAGCAGCCGCGGTAATACRDAG	
16S‐C8F^F^	AGAGTTTGATCMTGGCTCAG	Evaluation of a protocol for molecular broad‐range diagnosis of culture‐negative bacterial infections in clinical routine diagnosis. *J Appl Microbiol*. 2008 [3]
16S‐C535R^R^	CTTTACGCCCARTRAWTC	
16S‐CT535^T^	TNTTACCGCGGCTGCTGGCACG	
16S‐DF^F^	AGTTTGATC(A/C)TGGCTCAG	Detection of bacteria and fungi in blood of patients with febrile neutropenia by real‐time PCR with universal primers and probes. *J Infect Chemother*. 2015 [4]
16S‐DR^R^	GGACTAC(C/T/A)AGGGTATCTAAT	
16S‐DT^T^	CGTATTACCGCGGCTGCTGGCAC	
PLK1^F^	TACGGGAGGCAGCAGT	Droplet digital polymerase chain reaction for rapid broad‐spectrum detection of bloodstream infections. *Microb Biotechnol*. 2020 [5]
PLK2^R^	ATTACCGCGGCTGCT	
GRAM+ ^T^	CTAACCAGAAAGCCACGGCTAACTACGTG	
GRAM–^T^	TTACCCGCAGAATAAGCACCGGCTAAC	

## Appendix B (Informative) (see Table B1)

**TABLE B1:** Representative published universal primers and probes for fungi detection.

Primer/probe	Sequence (5′‐3′)	References
FungiQuantF	GGRAAACTCACCAGGTCCAG	FungiQuant: A broad‐coverage fungal quantitative real‐time PCR assay. *BMC Microbiology* 2012 [6]
FungiQuanR	GSWCTATCCCCAKCACGA	
FungiQuantT	TGGTGCATGGCCGTT	
B2FF	ACTTTCGATCGTAGGATAG	The Pathological Society of Great Britain and Ireland Detection of a wide range of medically important fungi by the polymerase chain reaction. *J Med Microbiol*. 1994 [7]
B4RR	TGATCGTCTTCGATCCCCTA	
18SIN3T	CTGAGAAACGGCTACCACAT	
18S‐AFF	ATTGGAGGGCAAGTCTGGTG	Detection of bacteria and fungi in blood of patients with febrile neutropenia by real‐time PCR with universal primers and probes. *J Infect Chemother*. 2015 [4]
18S‐ARR	CCGATCCCTAGTCGGCATAG	
18S‐ATT	TTCAACTACGAGCTTTTTAACTG	
ITS2FF	ATCGATGAAGAACGYAGC	Detection of fungal pathogens by a new broad range real‐time PCR assay targeting the fungal ITS2 region. *J Med Microbiol*. 2017 [8]
ITS2RR	TCCTCCGCTTATTGATATG	
ITS2TT	TACCCGCTGAACTTAAGCAT—	
FUNGIF	TCAATAAGCGGAGGAAAAGAAACC	Droplet digital polymerase chain reaction for rapid broad‐spectrum detection of bloodstream infections. *Microb Biotechnol*. 2020 [5]
FUNGIrAR	AGATGGAATTTACCACCCACTTAGA	
FUNGIrBR	AGATGGAATTTACCACCCGCTTGGA	
FungiProbeT	CGGCGAGTGAAGCGGSAARAGCT	

## Appendix C (Informative) Study Protocol for Determining the Limit of Detection (LOD)

**C.1 General**

The limit of detection (LOD) for a given detection procedure is determined under fixed experimental conditions (detection environment, reagents and instruments) for a specified sample type by testing gradient‐spiked samples. It is defined as the lowest spiked ratio that can be consistently detected with detection values significantly exceeding that of the unspiked background sample under these predetermined conditions.

**C.2 Experimental Design**

**C.2.1 Sample Preparation**

**C.2.1.1 Background Sample:**

Using batch samples from batches that have passed the sterility test specified in 1101 Part IV of the Chinese Pharmacopoeia as background samples [9].

**C.2.1.2 Positive Quantitative Reference Strains:**

Using the following reference strains specified in 1101 ‘Sterility Testing’ of Part IV of the Pharmacopoeia of the People's Republic of China [9].

*Staphylococcus aureus* [CMCC(B) 26 003]

*Pseudomonas aeruginosa* [CMCC(B) 10 104]

*Bacillus subtilis* [CMCC(B) 63 501]

*Clostridium sporogenes* [CMCC(B) 64 941]

*Candida albicans* [CMCC(F) 98 001]

*Aspergillus niger* [CMCC(F) 98 003]

*Escherichia coli* [CMCC(B) 44 102]

The recommended range for standard strains is (0.5–1.0) × 10^3^ CFU/unit. Due to potential differences in nucleic acid extraction efficiency among different bacterial species, it is recommended to conduct preliminary LOD studies for each standard strain individually. After obtaining preliminary data for each strain, design mixed spiked samples containing multiple strains based on their respective LOD values for confirmatory studies.

**C.2.1.3 Sample Aliquot:**

Collect an adequate quantity of test samples according to experimental requirements. Prepare samples at appropriate volumes/concentrations based on the extraction requirements of the selected nucleic acid extraction kit. Aliquot the prepared samples into a sufficient number of 1.5 mL microcentrifuge tubes. Prepare experimental and control group samples as described below.

**C.2.1.4 Experimental Group Samples:**

- **Test Samples: Use the aliquoted samples prepared in Step C.2.1.3**.
- **Positive Gradient Spiked Samples:**

Take one standard strain sample and resuspend it in 2 mL of bacterial resuspension solution, mixing gently.

Add gradient volumes of the resuspended bacterial solution (e.g., 10, 30μL, 100, 300, 600 μL, etc.) to the aliquoted samples prepared in Step C.2.1.3.

**C.2.1.5 Control Group Samples:**

- **Positive Extraction Control:** Use samples containing bacterial and fungal positive controls. Process these controls in parallel with the test samples, starting from sample treatment through subsequent detection steps.
- **Blank Extraction Control:** Perform extraction using sterile water or buffer instead of a sample. Process this control in parallel with the test samples through subsequent detection steps.
- **Positive Amplification Control:** Perform amplification using a nucleic acid solution with reliable target fragment amplification performance (e.g., bacterial and fungal genomic DNA or plasmid solutions containing the target amplification sequence).
- **Negative Amplification Control:** Perform amplification using sterile water or buffer as the template.

**C.2.2 Standard Strain Plate Counting**

Plate the remaining bacterial resuspension solution from Step C.2.1.4(b) at 100 μL per plate for colony counting. Include ≥ 5 replicate plates for counting.

Select culture plates and incubation temperatures according to 1105 ‘Microbial Limits Testing for Non‐Sterile Products: Microbial Enumeration Methods’ in Part IV of the Pharmacopoeia of the People's Republic of China [10].

Incubation times: Bacterial standard strains: ≤ 3 days. Fungal standard strains: ≤ 5 days

Calculate the spiking amount based on the plate counting results and volume ratios.

**C.2.3 Nucleic Acid Extraction and qPCR Detection**

Perform nucleic acid extraction and qPCR on experimental and control group samples according to the established detection procedure.

**C.2.4 Detection Experiment and Replication Validation**

**C.2.4.1 Parallel Detection Replicates:** Include ≥ 10 technical replicates per sample. Analyse and record the detection results.

**C.2.4.2 Batch‐to‐Batch Validation:** Perform the LOD experiment using at least three independent batches of samples following the same experimental protocol.

**C.2.5 LOD Confirmation Criteria**

**C.2.5.1 Prerequisites for LOD Confirmation:** Control group sample results must meet the quality control requirements outlined in Section 9.1.

**C.2.5.2 LOD Determination:** Compare test results and identify the minimum spiking amount where: all replicates across all batches show amplification Ct values < 30, and the Ct values are significantly lower than those of the blank extraction control and negative amplification control (*t*‐test, *p* ≤ 0.05).
